# “Golden” Tomato Consumption Ameliorates Metabolic Syndrome: A Focus on the Redox Balance in the High-Fat-Diet-Fed Rat

**DOI:** 10.3390/antiox12051121

**Published:** 2023-05-18

**Authors:** Giuditta Gambino, Giuseppe Giglia, Mario Allegra, Valentina Di Liberto, Francesco Paolo Zummo, Francesca Rappa, Ignazio Restivo, Filippo Vetrano, Filippo Saiano, Eristanna Palazzolo, Giuseppe Avellone, Giuseppe Ferraro, Pierangelo Sardo, Danila Di Majo

**Affiliations:** 1 Department of Biomedicine, Neuroscience and Advanced Diagnostics (BIND), University of Palermo, 90127 Palermo, Italy; giuseppe.giglia@unipa.it (G.G.); valentina.diliberto@unipa.it (V.D.L.); francescopaolo.zummo@unipa.it (F.P.Z.); francesca.rappa@unipa.it (F.R.); giuseppe.ferraro@unipa.it (G.F.); pierangelo.sardo@unipa.it (P.S.); danila.dimajo@unipa.it (D.D.M.); 2 Euro Mediterranean Institute of Science and Technology (IEMEST), 90139 Palermo, Italy; 3 Postgraduate School of Nutrition and Food Science, University of Palermo, 90100 Palermo, Italy; mario.allegra@unipa.it; 4 Department of Biological, Chemical and Pharmaceutical Sciences and Technologies (STEBICEF), University of Palermo, Viale delle Scienze, 90128 Palermo, Italygiuseppe.avellone@unipa.it (G.A.); 5 Dipartimento Scienze Agrarie, Alimentari e Forestali, Università degli Studi di Palermo, Viale delle Scienze Ed.4, 90128 Palermo, Italy; filippo.vetrano@unipa.it (F.V.); filippo.saiano@unipa.it (F.S.); eristanna.palazzolo@unipa.it (E.P.); 6 ATeN (Advanced Technologies Network) Center, Viale delle Scienze, 90128 Palermo, Italy

**Keywords:** tomato-based products, metabolic syndrome, HFD, antioxidant capacity, phytonutrients

## Abstract

Tomato fruits defined as “golden” refer to a food product harvested at an incomplete ripening stage with respect to red tomatoes at full maturation. The aim of this study is to explore the putative influence of “golden tomato” (GT) on Metabolic Syndrome (MetS), especially focusing on the effects on redox homeostasis. Firstly, the differential chemical properties of the GT food matrix were characterized in terms of phytonutrient composition and antioxidant capacities with respect to red tomato (RT). Later, we assessed the biochemical, nutraceutical and eventually disease-modifying potential of GT in vivo in the high-fat-diet rat model of MetS. Our data revealed that GT oral supplementation is able to counterbalance MetS-induced biometric and metabolic modifications. Noteworthy is that this nutritional supplementation proved to reduce plasma oxidant status and improve the endogenous antioxidant barriers, assessed by strong systemic biomarkers. Furthermore, consistently with the reduction of hepatic reactive oxygen and nitrogen species (RONS) levels, treatment with GT markedly reduced the HFD-induced increase in hepatic lipid peroxidation and hepatic steatosis. This research elucidates the importance of food supplementation with GT in the prevention and management of MetS.

## 1. Introduction

Tomato fruits *(Lycopersicon esculentum Mill.)* have increasingly grabbed attention as this food product, largely cultivated and consumed throughout the world, represents an invaluable source of bioactive compounds [1]. Noteworthy is that a wide plethora of nutritional substances are encountered in this food, i.e., antioxidants, such as flavonoids and naringenin, but also macronutrients, micronutrients, and organic and phenolic acids. Though the differential composition and quantity of discrete molecules depend on the cultivating conditions and could, hence, influence health-promoting activities, this still needs to be fully unveiled in terms of biochemical and nutritional characterization. In this context, “Golden Tomato” (GT) is a food product harvested at different degrees of ripeness and defined as “golden” due to the degree of coloring it possesses. A complete characterization in phytonutrients of this food product is deserved to fully explore its biological potential. As a matter of fact, previous research suggested a protective role for tomato-based products, modulating lipid profiles and positively influencing the development of cardiovascular diseases [2,3,4,5]. In particular, several clinical studies supported a role for different phytoconstituents of red tomato (RT) that can, for instance, improve the levels of antioxidant enzymes reducing lipid peroxidation rate in diabetic syndrome [6,7,8,9].

Metabolic syndrome (MetS) is a widely-diffused clustering of risk factors associated with obesity, cardiovascular disease, and alteration of oxidative status, conditions that could severely impact other comorbidities [10,11,12,13,14]. MetS is established if three or more of the following clinical conditions are present: hypertension, atherogenic dyslipidemia, increased visceral obesity, and hyperglycemia/insulin resistance [15,16]. From a mechanistic perspective, MetS development strongly relies on a vicious self-feeding cycle between chronic, low-grade inflammation and oxidative stress that predisposes individuals to cardiovascular diseases and type II diabetes. At the cellular level, oxidative stress consists of an increased production of reactive oxygen and nitrogen species (RONS) through the intervention of enzymatic, non-enzymatic, and/or mitochondrial pathways. In the background of the metabolic syndrome, NADPH oxidase emerges as a key ROS-producing enzyme [17]. Relevantly, RONS can interact with polyunsaturated fatty acids (PUFA), producing reactive lipid by-products (lipid peroxides and byproducts such as aldehydes) prone to interact with macromolecules such as proteins and DNA, leading to cellular dysfunction. At the same time, RONS overproduction modulates specific, intracellular kinase activities such as Jun N-terminal kinase and those related to NF-kB activation. These molecular events result in the increased phosphorylation of Ser/Thr residues of insulin receptor substrate (IRS-1) and lead to the impairment of both insulin signaling and glucose transport, frequently associated to MetS.

In animal models, MetS can be induced by employing a special diet regimen, i.e., a high-fat diet (HFD), that reproduces the complete clinical manifestations in terms of increased body weight, reduced food intake, glucose tolerance, and dyslipidemia [18,19,20]. Recently, our previous research revealed that specific systemic biomarkers of redox homeostasis are robustly predictive of the development of metabolic dysfunctions, strengthening the impact of oxidative-based alterations in MetS. A growing interest has arisen for the use of nutraceuticals contained in foods that can counteract MetS [21,22]. In this context, tomato stands out as a food of the Mediterranean diet representing an excellent source of nutrients and bioactive compounds, the concentrations of which are related to the prevention of chronic degenerative diseases such as cardiovascular disorders, cancer, and neurodegenerative diseases [11,23]. Numerous studies have already demonstrated the beneficial effect of tomato consumption on the lipid and glycemic profile [23,24]. Along these lines, we here evaluated the impact of the oral supplementation with GT, at an incomplete ripening stage, on the development of a multifactorial metabolic dysfunction, i.e., MetS, focusing on redox homeostasis. Since no studies so far have assessed the specific composition of GT as it is harvested at the ripening stage, we first investigated the different phytonutrients and antioxidant properties with respect to tomatoes harvested at full maturation. In detail, we characterized the food matrices of the tomato samples assessing the amount of phytonutrients present, but also the antioxidant properties via evaluation of radical scavenger activity for the purpose of discriminating between GT and RT. Secondly, we tested the impact of GT extracts in eventually counterbalancing MetS by exploiting a HFD rat model in order to evaluate the impact of this food product on oxidative-based impairments. Thus, we evaluated the influence of GT supplementation on altered body weight gain, glucose tolerance, lipid profile, and, even more importantly, on robust systemic and hepatic biomarkers of oxidative balance in MetS. This research could shed a novel light on the importance of food supplementation with GT in the prevention and management of MetS.

## 2. Materials and Methods

### 2.1. Treatment of Tomato Samples

The food matrix named “Golden Tomato” is the sample employed in the present research, which refers to a tomato harvested at the veraison stage and defined as “golden” on the basis of the degree of coloring it possesses. It is a tomato from the cultivar Brigade, grown in a sandy soil type with a northwest exposure and 650 m of altitude. Golden tomatoes were kindly provided by the manufacturer, Mr. Fabrizio Gioia (Company: “Azienda Agricola Fabrizio Gioia”, Montemaggiore Belsito, PA, Italy).

The nutritional characterization of the red and golden tomato samples was carried out by determination of the following parameters: macronutrients (proteins, lipids, and carbohydrates) and micronutrients (mineral salts). The degree of ripeness was established by means of the colorimetric analysis of the fruit and based on the following analytical parameters: pH, Brix degree, acidity, and polyphenolic content.

Both tomato samples, after harvesting, were divided into aliquots (~1 kg), freeze-dried and vacuum-preserved at −20 °C while waiting to be analyzed and used for animal treatment.

#### 2.1.1. Chemical–Physical Analysis of Tomato Samples

The tomato samples were subdivided according to colorimetric grade and certain chemical–physical parameters. The colorimetric analyses were carried out using a colorimeter (CR-400, Minolta corporation, Ltd., Osaka, Japan) based on the CIELAB color space, also referred to as L*a*b*, is a color space defined by the International Commission on Illumination (abbreviated CIE) in 1976. It expresses color as three values: L* for perceptual lightness and a* and b* for the four unique colors of human vision: magenta, green, blue and yellow. These components were used to calculate hue angle (h°) and chroma (C*) as

$$\begin{array}{c} h{}^{\circ}=180{}^{\circ}+arctan\left(b^{*}|a^{*}\right)\\ C^{*}=\sqrt{\left(a^{*2}+b^{*2}\right)}\\ h{}^{\circ}=180{}^{\circ}+arctan\;(b^{*}/a^{*})\;and\;C^{*}=(a^{*2}+b^{*2})1/2 \end{array}$$

following established procedures [25,26].

The basic analytical parameters characterizing the red and golden tomatoes and their different degree of ripeness were moisture, ash, pH, Brix degree, and acidity [Metodi ufficiali di analisi per le conserve vegetali—Parte generale, Supplemento ordinario alla Gazzetta Ufficiale n. 168 del 20 luglio 1989]. In particular, the pH was determined with the help of a pHmeter instrument, as is stated in the Italian regulations and the Official Methods of Analysis, AOAC.

#### 2.1.2. Determination of Phytonutrients in Golden and Red Tomatoes by HPLC System

The analysis of certain phytonutrients that could differentiate the red from the golden tomato involved a phase of extraction with an organic solvent from the food matrix, a phase of separation, and, successively, identification and quantification in the HPLC system. A total of 200 mg of powdered dried GT and RT samples were weighed and extracted with the addition of 20 mL of Tetrahydrofuran (THF). The samples were then processed with Ultra Turrax for about 30″ at 17,500 rpm, filtered with filter paper and, subsequently, with regenerated cellulose syringe filters (0.20 μm), and then loaded into autosampler vials and analyzed in HPLC.

For the HPLC analysis of tomato extracts, we used the UPLC-Q Exactive Orbitrap-HRMS system (Thermo Fisher Scientific™, Bremen, Germany) composed of a Dionex Ultimate 3000 liquid chromatograph coupled to a Q Exactive™ Plus Hybrid Quadrupole-Orbitrap™ Mass Spectrometer equipped with a heated electrospray ionization (HESI) ion source (detailed procedures are reported in Supplementary Materials).

#### 2.1.3. Determination of Total Polyphenolic Content and Antioxidant Properties

In general, the methods used to assess the antioxidant capacity of food can be divided according to their mechanism of action into two categories: methods based on hydrogen atom transfer (HAT) and those based on single-electron transfer (SET).

SET-based methods assess the ability of a compound defined as an antioxidant’s potential to yield an electron by reducing the acceptor compound, which can be a metal. This property is quantified by the color change observed when the oxidant is reduced. These methods include the Ferric Reducing Antioxidant Power (FRAP) and the Folin–Ciocalteu methods. HAT-based methods measure the classical ability of an antioxidant to quench free radicals by hydrogen donation. The Crocin bleaching assay (CBA) is included among the HAT methods [27,28]. To evaluate the antioxidant properties of tomato samples, we used FRAP and Folin–Ciocalteu as SET methods and the Crocin bleaching assay as a HAT method. The analyses were performed on the methanolic extract obtained from fresh tomatoes (detailed procedures are reported in Supplementary Materials). The results were expressed as mean ± standard error of the mean (S.E.M.) of three replicates.

### 2.2. Animals

Male Wistar rats (4-week old) weighing 240–260 g were provided by Envigo S.r.l. They were housed two per cage and maintained on a 12 h on/off light cycle (8:00–20:00 h) at a constant temperature (22–24 °C) and humidity (50 ± 10%). During the acclimation period, animals were first fed with a standard chow diet providing 3.94 kcal/g and then divided into two homogenous groups with balanced weight. These groups were fed with standard laboratory food (NPD: normal pelletized diet, code PF1609, certificate EN 4RF25, Mucedola, Milan, Italy) or fed with HFD food with 60% of energy coming from fats (code PF4215-PELLET, Mucedola, Milan, Italy) in order to induce MetS, as assessed following criteria already established by previous literature [19,20]. Detailed description of the composition of the diet is included in Table 1, as in our previous study [20]. All rats had free access to food and water. Prior to starting the special diet, all animals were weighed. The experiment involved three stages—T0, T1, and T2—individuated according to procedures described in detail in Supplementary Materials. Animal care and handling throughout the experimental procedures were in accordance with the European Communities Council Directive (2010/63/EU). The experimental protocols were approved by the animal welfare committee of the University of Palermo, authorized by the Ministry of Health (Rome, Italy; Authorization Number 14/2022-PR), and conducted following the ARRIVE guidelines.

#### Experimental Groups

Each experimental group consisted of *n* = 6 animals, except for one group (NPD, *n* = 4). At T0, animals were initially subdivided in NPD or HFD on the basis of the diet administered for 8 weeks until induction of MetS. At T1, once MetS induction was verified, animals were divided into 4 groups according to the type of diet (NPD and HFD) and treatment administered (golden tomato or red tomato) until T2, which was reached 4 weeks after T1. In particular, the normal control was fed normal diet feed (NPD) until T2 and the second one (HFD group), representing the MetS control, was fed with the HFD diet throughout the trial (from T0 to T2). The control NPD and HFD groups were subjected i.p. to the same stress conditions as the treated group, since they received during the last month of the experiment, from T1 to T2, a volume of vehicle equal to the tomato solution administered to the treated groups. Furthermore, one group (HFD/GT) was treated 1 mL daily with golden tomato (GT) in the last month of the trial (T1-T2). Lastly, a group of rats was orally treated with red tomato (HFD/RT) at full maturation, at the same dose and under the same experimental conditions as HFD/GT group for 1 month, in order to verify eventual specific effects of red tomato on the redox homeostasis biomarkers of MetS.

### 2.3. Preparation of the Orally Administered Tomato Solutions

The amount of tomato administered was 200 mg/kg body weight, corresponding to a daily intake of 300 g for a man with an average weight of 70 kg. The dose was established on the basis of valid toxicity tests for red tomatoes in the literature [29,30] and considered to be over the minimum dose exerting an eventual biological effect when translated from animal studies to humans [31].

The dose was obtained by dissolving 50 mg of freeze-dried fresh tomato in solution with 50 mL of water. The volume of golden tomato solutions orally-administered daily using a syringe was 1 mL. The groups not receiving the tomato solutions took the same volume (1 mL) of plain water. No animals showed signs of toxicity or intolerance during the treatments.

### 2.4. Biometric, Biochemical, and Oxidative Homeostasis Parameters Induced by MetS

At the T2 time point, the effect of treatments on the experimental groups was evaluated on the MetS-induced alterations in terms of biometric, biochemical, and oxidative homeostasis parameters. At the end of the experimental procedures, all animals were sacrificed using 2% isoflurane anesthesia followed by cervical dislocation in accordance with authorized procedures. Plasma samples were collected for subsequent analyses to evaluate lipid homeostasis, oxidative stress parameters, and plasma antioxidant status. Hepatic samples were also collected for malondialdehyde (MDA), RONS, and GSH determination as well as for histological evaluations.

#### 2.4.1. Body Weight Gain

Body weight gain was evaluated at T2 for all animals after 4 weeks of nutritional treatments, calculating the Delta Body Weight (ΔBW) by subtracting the final rat weight from the initial weight recorded at T0.

#### 2.4.2. Glucose and Lipid Homeostasis assays

Glucose Tolerance Test (GTT), a diagnostic tool for diabetes and an indicator of metabolic efficiency and insulin resistance, was conducted at T2 following established procedures [20,32] to evaluate the effect of nutritional treatments on glucose metabolism in MetS. To assess the effect of GT supplementation on lipid homeostasis in MetS, after sacrifice, blood samples of each animal were collected by cardiac puncture. Detailed procedures are described in our previous paper [20]. In the plasma samples, triglycerides (TG), total cholesterol (TC), low-density lipoprotein cholesterol (LDL), and high-density lipoprotein cholesterol (HDL) concentrations were quantified by commercial kits using the Free Carpe Diem device (FREE^®^ Carpe Diem; Diacron International, Grosseto, Italy). The data are expressed in mg/dL.

#### 2.4.3. Oxidative Stress Parameters and Plasma Antioxidant Status

Plasma redox balance was assessed using Diacron kits following detailed procedures already published [20]. To assess the prooxidant status, the dROM (Reactive Oxygen Metabolites, primarily hydroperoxides) and the LP-CHOLOX test were carried out, the former assessing the levels of hydroperoxyl free radicals and the latter the levels of circulating lipid peroxides and, in particular, oxidized cholesterol. In the plasma samples, hydroperoxides, lipoperoxides, and oxidized cholesterol were measured by commercial kits using the Free Carpe Diem device (FREE^®^ Carpe Diem; Diacron International, Grosseto, Italy). Data from dROM tests are expressed in arbitrary units, namely, Carratelli units (UCARR). The normal range of the test results was 250–300 U.CARR (Carratelli Units), where 1 U.CARR corresponds to 0.08 mg/dL of H_2_O_2_ [33]. In the LP-CHOLOX test, LP-CHOLOX (lipoperoxides and oxidized cholesterol) levels are detected based on peroxides’ ability to facilitate the oxidation of Fe^2+^ to Fe^3+^, which binds to an indicator mixture forming a colored complex detected by a spectrophotometer at 505 nm [34,35]. The results are expressed in mEq/L.

As for the plasma antioxidant status, the “BAP” test (Biological Antioxidant Potential) measures substances of an exogenous nature (ascorbate, tocopherols, carotenoids, and bioflavonoids) and substances of an endogenous nature (bilirubin, uric acid, and proteins) in plasma that have antioxidant potential and are capable of counteracting radical species. The analysis was performed using the Diacron kit by taking spectrophotometric readings at a wavelength of 505 nm as reported in a previous work and expressing the results as mmol/L [36]. Furthermore, the SHp test was used for evaluation of thiol groups to assess the reducing properties of tomato extracts that can counteract the oxidation of thiol groups and shift the balance in favor of reduced forms.

#### 2.4.4. MDA Assay

Evaluation of MDA levels in liver homogenates was performed according to Ohkawa et al. [37]. Briefly, the reaction mixture contained 0.2 mL of whole homogenate, 0.2 mL of 8.1% sodium dodecyl sulphate (SDS), 1.5 mL of acetic acid solution adjusted at pH 3.5 with NaOH, and 1.5 mL of 1% thiobarbituric acid (TBA) aqueous solution. The mixture was finally made up to 4.0 mL with distilled water and heated at 95 °C for 60 min. After cooling with tap water, 1.0 mL of distilled water and 5.0 mL of a n-butanol/pyridine solution (15/1, *v*/*v*) were added, and the mixture was shaken vigorously. After centrifugation at 4000 rpm for 10 min, the absorbance of the organic layer was measured at 532 nm. MDA levels were expressed as nmol MDA/g tissue, using 1,1,3,3,tetramethoxypropane as an external standard.

#### 2.4.5. RONS Assay

RONS levels were detected in liver homogenates using 2′,7′dichlorodihydrofluorescein diacetate (H_2_DCF-DA) as previously reported [38]. Briefly, the whole homogenate was centrifuged at 3500 rpm for 10 min at 4 °C and 100 µL of the supernatant was mixed with 5 µL of H_2_DCF-DA (final concentration 10 µM). The mixture was incubated for 30 min at 37 °C protected from light and the fluorescence intensity was detected at 490 nm (excitation) and 540 nm (emission) by using a plate reader.

#### 2.4.6. GSH Measurements

Hepatic GSH/GSSG levels were measured in the whole homogenate by employing a glutathione colorimetric assay kit according to the manufacturer’s instructions (Invitrogen, Milan, Italy).

### 2.5. Histological Analyses

Hepatic samples were immediately stored in paraformaldehyde for 48 h, then were moved to a 20% PBS/sucrose solution, and, after 1 week, to 10% PBS/sucrose solution. At last, the solution was removed and samples were stored at −80°. Liver tissue sections (5 µm) were obtained from cryostat and stained with hematoxylin and eosin. Following staining, the slides were observed with an optical microscope (Microscope Axioscope 5/7 KMAT, Carl Zeiss, Oberkochen, Germany) connected to a digital camera (Microscopy Camera Axiocam 208 colour, Carl Zeiss). For the steatosis evaluation, a semiquantitative analysis was performed by two independent observers in a high-power field (HPF) (magnification 400×) and repeated for 10 HPFs.

### 2.6. Statistical Analyses

Statistical analysis was performed by using GraphPad Prism 9.02 (San Diego, CA, USA). Analyses of antioxidant composition between GT and RT samples were performed by an unpaired Student’s *t*-test. Plasma glucose levels (GTT) were analyzed via a two-way repeated measures (RM) ANOVA, followed by Bonferroni post hoc test for significant differences for within- and between-subject comparisons, considering the effect of “time”, “diet”, and their interaction in the experimental groups. Values of ΔBW, GTT, TG, TC, AUC, MDA, and RONS levels and histological evaluations in liver were compared by a one-way ANOVA test followed by Bonferroni post hoc evaluations for differences between means and represented by scattered bar graphs, in which at least 4 animals per group were included for evaluation. Differences were considered significant when *p* < 0.05. The statistical power (g-power) was considered only if >0.75 and the effect size if >0.40. The results are presented as the mean ± standard error of the mean (S.E.M.), apart from GTT values at 0 and 120 min presented as box and whiskers plots.

## 3. Results

### 3.1. Analytical, Nutritional, and Antioxidant Composition of Tomato Food Matrices

The different ripening time determines the different phytonutrient composition and antioxidant properties of the tomato, making the red and golden tomato two different food matrices despite coming from the same cultivar and the same production conditions. In particular, the compounds identified in the two food matrices, together with the nutritional properties, micronutrient and organic acid composition are indicated in Supplementary Tables S1–S4. The chemical–physical characteristics used to distinguish the two groups of red (RT) and golden (GT) tomatoes are shown in Table 2.

Based on the colorimetric analysis, four different colourings were considered with varying color gradations, evidenced by the C* and h° parameters calculated with the specific equations given in the text (Section 2.1.1), from the red fruits (C* = 25.11 and h° = 115.58) in a full ripening state to colorings of decreasing intensity up to the green fruits. Among these, only green (C* = 32.08 and h° = 264.56), pre-veraison (C* = 30.01 and h° = 264.72) and veraison tomatoes (C* = 30.07 and h° = 264.68) were used to produce “golden” sample GT.

**Figure 1:**
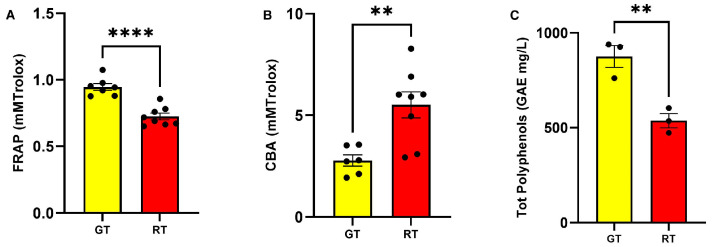
Analyses of antioxidant properties of golden tomato and red tomato (GT and RT) samples. Evaluations using (**A**) Ferric Reducing Antioxidant Power (FRAP, mM/trolox), (**B**) Crocin Bleaching Assay (CBA, mM/trolox), and (**C**) total polyphenolic content (GAE mg/L) were reported and significant differences following unpaired *t*-test between GT and RT are indicated as (****) *p* < 0.0001 and (**) *p* < 0.01.

As described in Table 2, tomatoes at veraison stage (GT) have a slightly lower water content in absolute value than ripe tomatoes (RT), making all macronutrients slightly more concentrated. Furthermore, as might be expected, GT has a higher degree of acidity and lower pH than RT. From a nutritional viewpoint, GT and RT show minor differences in macronutrients and energy value provided (data reported in supplementary material) even if the reduced degree of ripeness makes the tomato poorer in vitamin C by 45% and in pro-vitamin A, expressed as beta-carotene, by 69%, as shown in Table 3. Regarding the polyphenolic content and antioxidant properties of tomato samples, the different antioxidant properties of GT and RT samples were evaluated via HAT and SET methods. GT exhibits a superior reducing power compared to RT, and this can be observed from results obtained by FRAP and Folin–Ciocalteu methods. In detail, the FRAP assay revealed a significant increase in GT versus RT samples (t = 6.18, df = 13, *p* < 0.0001, Figure 1A). Conversely, the CBA assay revealed a significant increase in RT versus GT samples (t = 3.48, df = 12, *p* = 0.0045, Figure 1B). Lastly, the analysis of total polyphenolic content revealed that GT samples contained significantly higher levels of polyphenols as in Figure 1C (t = 4.923, df = 4, *p* = 0.0079). The antioxidant properties outlined in GT and RT samples could be ascribed to the differential composition in phytonutrients as listed in Table 2. In particular, we observed that, in the GT, the levels of naringenin and chlorogenic acid are 57% higher for the former and 81% higher for the latter, respectively, which stand out compared to RT. Meanwhile, RT is characterized by an increased content in lycopene and beta-carotene.

### 3.2. Effects of GT Treatment on Body Weight in MetS

**Figure 2:**
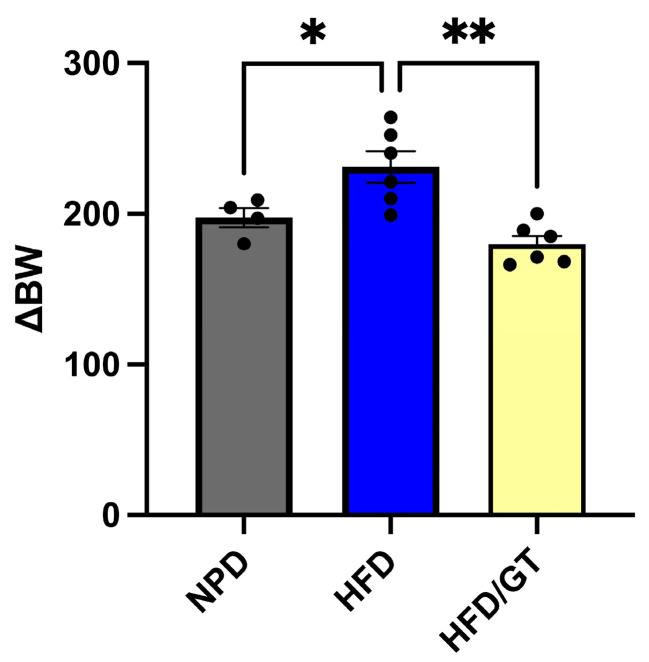
Variation of body weight gain (ΔBW) versus the initial weight in HFD/GT, HFD, and NPD experimental groups. Statistical significance by one-way ANOVA followed by post hoc Bonferroni is indicated as (**) for *p* < 0.001 versus HFD and (*) for *p* < 0.005 versus NPD.

At T2, after 4-week treatment with GT and RT, ΔBW was compared via one-way ANOVA followed by Bonferroni post hoc test. Significant differences in body weight were found in HFD/GT that reduced their weight gain versus HFD and versus NPD in MetS (F_(2,13)_ = 3.17, *p* = 0.0014, g-power: 1.307; effect size: 0.769; Figure 2). In detail, following treatment with GT, rats reached a mean % weight increase relative to initial weight of 78.75 ± 11.91 with respect to HFD (90.35 ± 10.74%g).

### 3.3. Effects of GT Treatment on Glucose and Lipid Homeostasis in MetS

**Figure 3:**
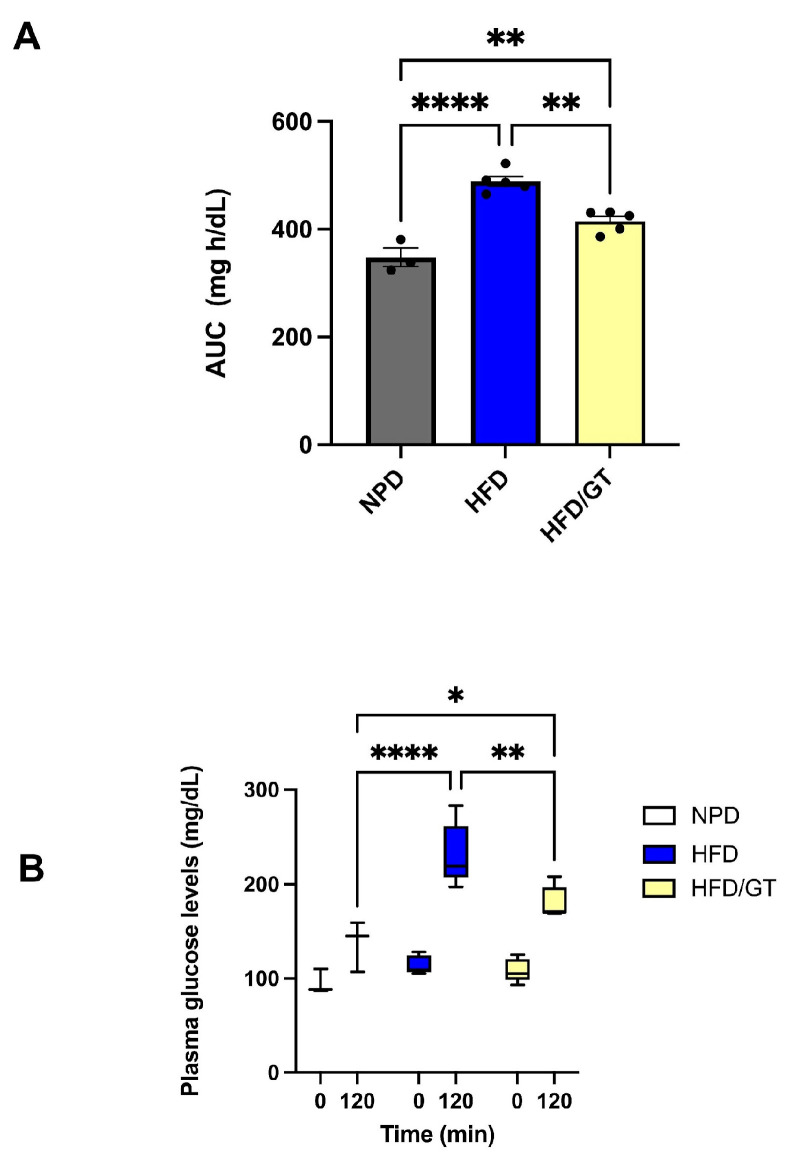
GTT test in experimental groups at the end of experimental procedures. (**A**) Area under the curve (AUC). Plasma glucose levels (mg/dL) per unit of time (h) difference between HFD/GT, HFD and NPD groups. (**B**) Glucose levels in GTT. Plasma glucose level (mg/dL) differences between groups after 0 and 120 min of GTT. Statistical significance by one-way ANOVA followed by post hoc Bonferroni is indicated as (****) *p* < 0.0001, (**) *p* < 0.01, and (*) *p* < 0.05.

The effects of nutritional treatments in MetS were tested on glucose homeostasis. To this purpose, GTT test was performed at the end of nutritional treatments and outlined significant differences between experimental groups. To begin with, we evaluated the AUC, as described in the methods section, by one-way ANOVA followed by a Bonferroni post hoc test that highlighted a significant reduction in HFD/GT group versus HFD and a significant increase versus NPD (F_(2,10)_ = 37.43, *p* < 0.0001, g-power: 0.819; effect size: 2.33; Figure 3A), suggesting an improvement in glucose tolerance tests following GT supplementation, though not returning to baseline. Besides this, a two-way RM ANOVA followed by a Bonferroni post hoc test performed on GTT at 0 and 120 min revealed marked differences in plasma glucose levels for time (F_(1,10)_ = 119.8, *p* < 0.0001), diet (F_(2,10)_ = 12.01, *p* = 0.0022), and their interaction (F_(2,10)_ = 9.44, *p* = 0.0050) in HFD/GT compared to HFD and NPD groups (Figure 3B). In accordance with previous data [39], no significant differences are observed between groups in fasting glucose levels while there are different trends over time after i.p. glucose administration. In particular, a significant reduction in plasma glucose was evidenced by post hoc Bonferroni tests due to GT treatment, since the HFD/GT group at 120 min reached lower values versus HFD. However, glucose tolerance was ameliorated but not compensated, since the HFD/GT group was still significantly higher than NPD, not returning to baseline.

As for the evaluation of lipid homeostasis at T2, we assessed the plasma concentrations of TG, TC, LDL, and HDL in the experimental groups. On the one hand, the one-way ANOVA performed on the plasma levels of TG did not point out significant differences between groups, as shown in Table 4. On the other hand, the plasma levels of LDL analyzed by one-way ANOVA were remarkably decreased following GT treatment in the HFD/GT group with respect to HFD, and returned to basal values that were not statistically different than the NPD group (F_(2,13)_ = 12.59, *p* = 0.0009; g-power: 0.769; effect size: 1.30; Table 4). Total cholesterol was markedly increased in HFD/GT rats versus HFD and NPD group (F_(2,11)_ = 16.39, *p* = 0.0005; g-power: 0.937; effect size: 1.688; Table 4). Importantly, HDL cholesterol was increased by GT treatment in HFD rats reaching significantly higher levels with respect to HFD and NPD groups (F_(2,11)_ = 20.84, *p* = 0.0002; g-power: 0.801; effect size: 1.358, Table 4).

### 3.4. Effects of GT on Plasma Redox Homeostasis Biomarkers in MetS

The antioxidant and prooxidant status was evaluated at time T2, after nutritional treatment, on plasma samples from all the experimental groups to explore the plasma redox balance in metabolic syndrome. Statistical analyses revealed that GT markedly modulated the antioxidant capacity of MetS animals.

**Figure 4:**
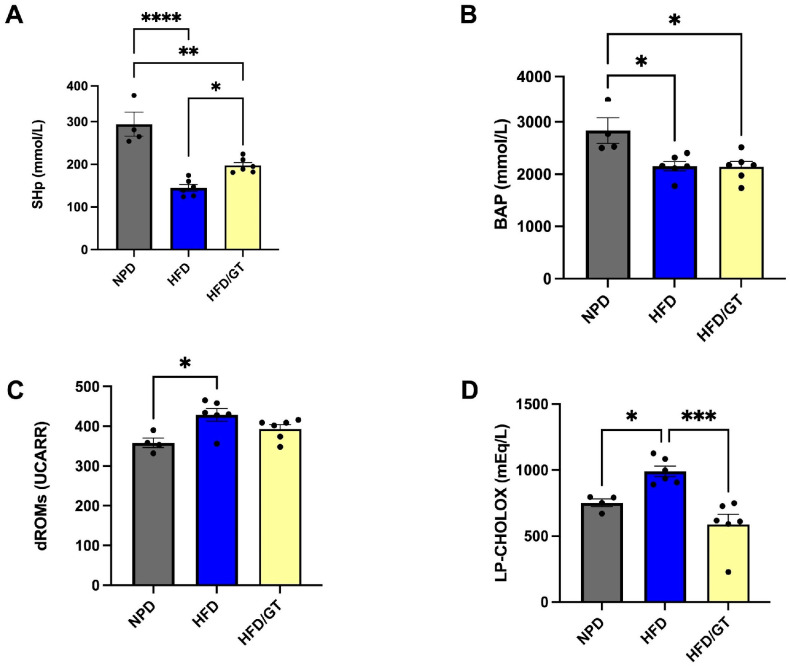
Plasma redox homeostasis biomarkers between HFD/GT, NPD, and HFD groups at the end of experimental procedures. Antioxidant status evaluated by (**A**) SHp test for thiol group levels (mmol/L) and (**B**) Biological Antioxidant Potential (BAP test) levels (mmol/L). Prooxidant status evaluated by (**C**) dROM test for differences in ROM (primarily hydroperoxide) levels (UCARR) and (**D**) LP-CHOLOX test for differences in LP-CHOLOX (lipoperoxides and oxidized cholesterol) levels (mEq/L). Statistical significance of Bonferroni post hoc tests are indicated for (*) *p* < 0.05, (**) *p* < 0.01, (***) *p* < 0.001 and (****) *p* < 0.0001, as represented in the graphs.

In particular, a one-way ANOVA followed by post hoc test was conducted on mean values of SHp in HFD/GT that are higher than HFD, though still significantly reduced versus NPD group (F_(2,13)_ = 27.09, *p* < 0.0001; g-power: 0.972; effect size: 1.869; Figure 4A). BAP values were reduced in both HFD/GT and HFD groups versus NPD (F_(2,13)_ = 7.22, *p* = 0.0078; g-power: 0.905; effect size: 0.793; Figure 4B). Furthermore, statistical significance emerged from the analysis conducted on mean values of the prooxidant status, i.e., dROM and LP-CHOLOX levels. In detail, dROM levels were modified among the experimental groups, though post hoc analysis revealed a non-significant reduction induced by GT treatment versus HFD (F_(2,13)_ = 6.15, *p* = 0.0132; g-power: 0.923; effect size: 0.809; Figure 4C).

Lastly, LP-CHOLOX levels were significantly reduced in the HFD/GT group versus HFD, returning to baseline since non-significant differences emerged with the NPD group (F_(2,13)_ = 13.63, *p* = 0.0006; g-power: 0.776; effect size: 1.318; Figure 4D).

### 3.5. Effects of GT on Hepatic Steatosis

**Figure 5:**
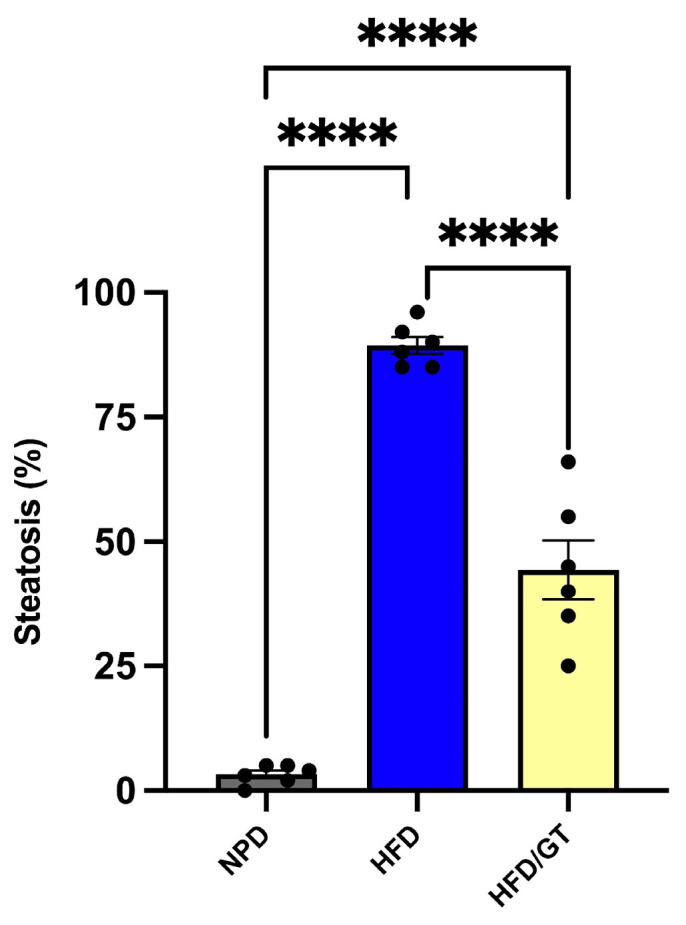
Histological evaluation of hepatic steatosis. Differences in hepatic steatosis (%) between NPD, HFD, and HFD/GT groups. **** for *p* < 0.0001.

**Figure 6:**
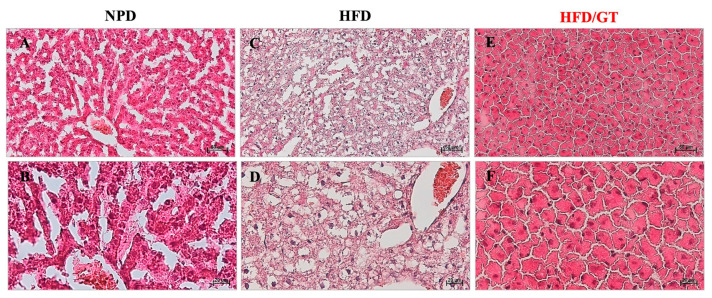
Histological features of liver tissue of experimental groups. Representative images of hematoxylin and eosin staining of liver tissue ((**A**,**B**): NPD; (**C**,**D**): HFD; (**E**,**F**): HFD/GT). (**A**,**C**,**E**): magnification 200×, scale bar 50 µm. (**B**,**D**,**F**): magnification 400×, scale bar 20 µm.

The histological evaluation performed on liver samples of the control NPD group showed an almost absent steatosis (average percentage of 3.2 ± 0.8) compared to the cases of the HFD group in which steatosis was found to be high (average percentage of 89.3 ± 1.7). In HFD liver tissue, macrovesicular steatosis with large lipid droplets was predominantly observed. In the liver samples of the GT group, the steatosis was microvesicular with an average percentage of 44.33 ± 14.5. Statistical evaluation by one-way ANOVA showed a significant decrease in the percentage of steatosis in HFD following GT supplementation versus HFD, though it was still significantly higher than NPD (F_(2,12)_ = 6.59, *p* = 0.011; g-power: 0.99; effect size: 5.07; Figure 5 and Figure 6).

### 3.6. Effects of GT on MetS-Induced Hepatic Oxidative Stress

**Figure 7:**
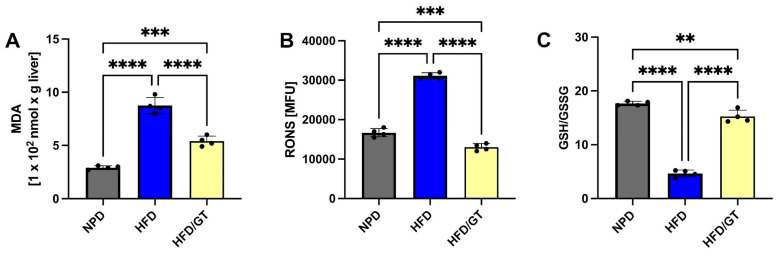
Levels of hepatic oxidative stress in HFD/GT, NPD, and HFD groups at T2. (**A**) Malondialdehyde (MDA) levels, (**B**) reactive oxygen and nitrogen species (RONS) levels, and (**C**) GSH/GSSG levels. Statistical significance by one-way ANOVA followed by post hoc Bonferroni is indicated as **** for *p* < 0.0001, *** for *p* < 0.001 and ** for *p* < 0.01.

As shown in Figure 7A, the statistical analyses performed by one-way ANOVA on MDA levels in the liver showed significant differences between groups. In particular, GT significantly reduced the HFD-induced oxidative stress in the same tissue in comparison with the HFD group, though basal NPD values were not restored (F_(2,9)_ = 124.2, *p* < 0.0001; g-power: 0.99; effect size: 5.19; Figure 7A).

The evaluation of RONS in the liver highlighted that the treatment with GT managed to recover the oxidative status induced by HFD by reducing hepatic RONS levels. In particular, GT was able to reduce RONS levels even in comparison with the NPD group as shown by post hoc significance in Figure 7B (F_(2,9)_ = 436.3, *p* < 0.0001; g-power: 1; effect size: 8.61). Finally and in accordance with this evidence, GT treatment also ameliorated the ratio of GSH/GSSG in the liver by significantly increasing its levels, although not to the control levels (Figure 7C, F_(2,9)_ = 289.2, *p* < 0.0001; g-power: 1; effect size: 7.561).

### 3.7. Effects of RT on MetS-Induced Systemic and Hepatic Oxidative Stress

The HFD/RT group of rats was employed in this study to unveil its specific effect on redox homeostasis in our experimental model. Therefore, we focused on the assessment of systemic and hepatic oxidative stress in MetS following nutritional treatment with RT at full maturation.

**Figure 8:**
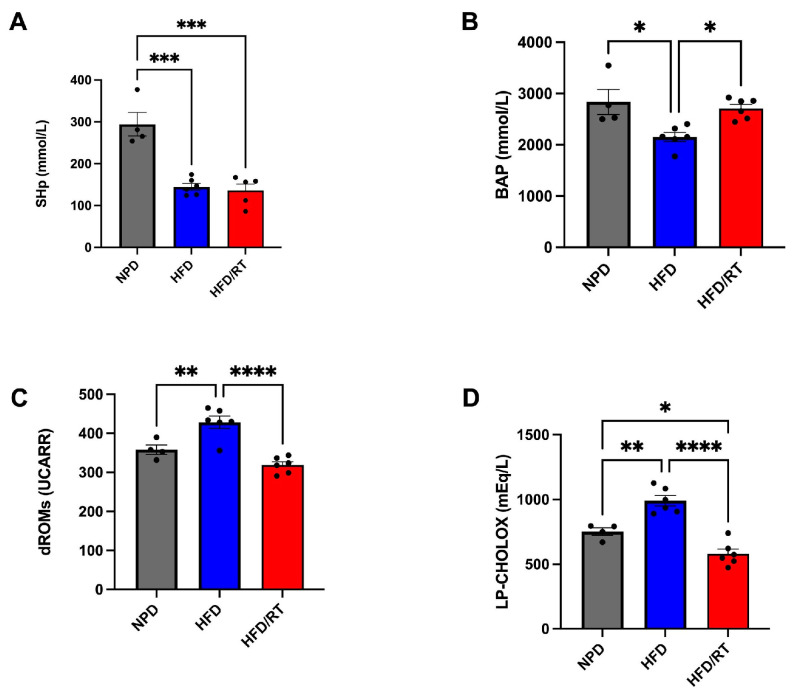
Plasma redox homeostasis biomarkers between HFD/RT, NPD, and HFD groups at the end of experimental procedures. Antioxidant status evaluated by (**A**) SHp test for thiol group levels (mmol/L) and (**B**) Biological Antioxidant Potential (BAP test) levels (mmol/L). Prooxidant status evaluated by (**C**) dROM test for differences in ROM (primarily hydroperoxides) levels (UCARR) and (**D**) LP-CHOLOX test for differences in LP-CHOLOX (lipoperoxides and oxidized cholesterol) levels (mEq/L). Statistical significance of Bonferroni post hoc tests are indicated for (*) *p* < 0.05, (**) *p* < 0.01, (***) for *p* < 0.001 and (****) *p* < 0.0001, as represented in the graphs.

To begin with systemic biomarkers, we discovered that RT treatment does not manage to rescue SHp values versus HFD as analyzed by a one-way ANOVA followed by a Bonferroni post hoc analysis (Figure 8A). In contrast, RT treatment significantly increases BAP values versus HFD, returning to the basal values of NPD group (F_(2,13)_ = 8.01, *p* = 0.0054; g-power: 0.986; effect size: 0.968; Figure 8B). As for the evaluation of the prooxidant status, statistical significance emerged from the analysis conducted on mean values of dROM and LP-CHOLOX levels. In detail, dROM levels were markedly reduced by RT treatment versus HFD (F_(2,13)_ = 21.02, *p* < 0.0001; g-power: 0.995; effect size: 1.72; Figure 8C) and LP-CHOLOX levels were significantly reduced in HFD/RT group versus HFD and also lower than the NPD group (F_(2,13)_ = 32.79, *p* < 0.0001; g-power: 0.993; effect size: 2.15; Figure 8D).

**Figure 9:**
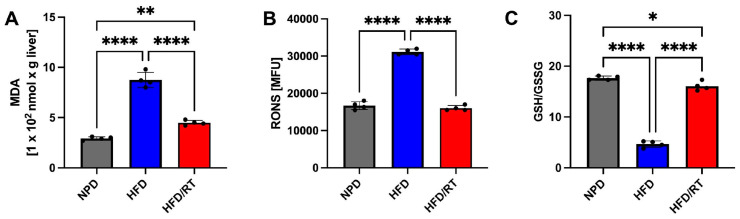
Levels of hepatic oxidative stress in HFD/RT, NPD, and HFD groups at T2. (**A**) Malondialdehyde (MDA) levels, (**B**) reactive oxygen and nitrogen species (RONS) levels, and (**C**) GSH/GSSG levels. Statistical significance by one-way ANOVA followed by post hoc Bonferroni is indicated as (*) for *p* < 0.05, (**) for *p* < 0.01 and (****) for *p* < 0.0001.

Regarding hepatic biomarkers of oxidative stress, our outcomes revealed that RT at full maturation is able to significantly reduce the HFD-induced MDA levels in hepatic tissue in comparison with the HFD group, though not restoring to basal NPD values (F_(2,9)_ = 163.4, *p* < 0.0001; g-power: 0.999; effect size: 6.26; Figure 9A). The evaluation of RONS in the liver highlighted that the treatment with RT managed to recover the oxidative status induced by HFD by reducing hepatic RONS levels and restoring basal NPD values (F_(2,9)_ = 399.5, *p* < 0.0001, g-power: 1; effect size: 8.30; Figure 9B). Lastly, GSH levels in RT-treated rats were higher than the HFD group, but still significantly different from control NPD rats (F_(2,9)_ = 434.8 *p* < 0.001; g-power: 1; effect size: 9.04; Figure 9C).

## 4. Discussion

GT is a food product harvested at an incomplete ripening stage and has a different nutritional and phytonutrient composition with respect to red tomato at full maturation. Not surprisingly, the two food matrices that we here analyzed for the first time differ to a great extent. To begin with, we revealed the higher acidity of GT samples compared to RT, which could account for a different bioavailability of phytonutrients [40] and for a different aggregation state of the polyphenolic compounds, which may explain the different antioxidant properties found in GT and RT food matrices. Indeed, we revealed the different phytonutrient composition that could be responsible for the higher antioxidant capacities of the GT in terms of reducing power compared to the RT. However, RT shows better radical scavenger activity than the GT evaluated by CBA. Among the phytonutrients, we demonstrated that GT has a higher naringenin and chlorogenic acid content than RT, which could be of striking importance since in vitro studies supported the beneficial effects of naringenin and of chlorogenic acid on MetS [23,41,42].

In the light of the intriguing antioxidant properties emerged by our evaluation of the GT food matrix, we orally administered GT extracts in an HFD rat model of MetS in order to further explore a putative protective role. Indeed, the HFD model was previously demonstrated to trigger an oxidative-dependent metabolic dysfunction that represents an undoubtedly valid model for the assessment of GT properties [20,43]. Our data revealed that one month of GT oral supplementation managed to reduce body weight gain in HFD rats. GT supplementation also counteracted the deleterious effect of the HFD on glucose tolerance. Nevertheless, the GT supplementation is able to ameliorate the glucose tolerance, but not to restore it to the basal values of the NPD group. Similarly to GT, some authors have shown that supplementation of naringenin or chlorogenic acid can improve but not normalize the manifestations of glucose tolerance [42,44,45]. Indeed, chlorogenic acid has anti-diabetic and anti-obesity properties by reducing glucose absorption in the small intestine through inhibition of the enzyme glucose-6-phosphate translocase, inhibiting the hepatic enzyme glucose-6-phosphatase, and increasing phosphorylation of AMP-activated protein kinase [46,47].

Noteworthy is that GT also reduced LDL cholesterol and increased HDL cholesterol versus both HFD and NPD rats. The same effect observed in our study was shown by administering 10 mg/Kg of chlorogenic acid to rats with hypercholesterolemia induced by the HFD diet [48]. This cholesterolemic-lowering effect could be due to the inhibitory action of naringenin and of chlorogenic acid on the enzyme HMG-CoA reductase already demonstrated [49,50,51]. On top of this, chlorogenic acid influences lipid metabolism by modulating the transcription of genes coding for lipogenic enzymes such as fatty acid synthase and acetyl-CoA carboxylase. In particular, it appears to induce down-regulation of LXR𝛼 and up-regulation of PPAR𝛼 [52].

As for the effect of GT supplementation on altered plasma biomarkers of antioxidant defenses in MetS, we here revealed that GT markedly modulated the systemic antioxidant capacity in vivo in the HFD model. The different composition in phytochemicals of the two food matrices allows to justify the behavior on plasma antioxidant status observed after administration of GT. In particular, the oral administration of GT to HFD rats was able to enhance SHp levels. GT is, in fact, composed of chlorogenic acid and naringenin, molecules with high reducing power, hence explaining the effect observed on thiol groups. Furthermore, the influence of GT supplementation on SH groups is supported by the result obtained in terms of reducing power, measured by FRAP in GT. We previously uncovered that SHp values negatively correlate with lipid profile biomarkers in MetS [20], thus showing that GT supplementation resets—in favor of thiolic groups—the MetS-induced shifted balance towards disulfide compounds. This could be due to a GT-mediated compensation that increases antioxidant defenses to counteract the excessive free radicals. It could appear counterintuitive that GT treatment did not manage to rescue BAP levels in HFD rats, though it further supports the specific effect induced by the reducing power of GT. In accordance with an improved antioxidant status, the oxidative biomarkers, i.e., dROMs and LP-CHOLOX, were powerfully decreased by GT supplementation in HFD rats, showing a better protection from plasmatic lipid peroxidation products. In detail, GT extracts reduced both LP-CHOLOX levels and, not significantly, dROMs values, though basal levels of normally fed rats were not restored. The protective effect of GT supplementation on plasma lipoperoxidation and the plasma antioxidant barrier could be ascribed to naringenin, whose ability to reduce lipid peroxidation and normalize antioxidant defenses by increasing the activity of antioxidant enzymes in the liver has been demonstrated in the literature [41]. Reduced plasmatic levels of hydroperoxides and lipoperoxides by GT supplementation are thought to well-correlate with the improved glucose profile revealed by our study.

Relevantly, not only does GT supplementation reduce systemic oxidative stress but also significantly counteracts the HFD-induced hepatic production of RONS. Interestingly, these species have been shown to enhance MDA levels in the liver [53]. This reactive aldehyde has been demonstrated to irreversibly form adducts with macromolecules, modifying cell function and contributing to MetS development [53,54,55]. Consistently with the reduction of hepatic RONS levels, treatment with GT also markedly reduced the HFD-induced increase in hepatic lipid peroxidation and increased the GSH/GSSG ratio in the liver. These results are in line with previously reported evidence showing how RT supplementation has been demonstrated to exert powerful, anti-oxidative effects able to counteract the onset and development of several pathological conditions in humans, including MetS [56]. Notwithstanding the fact that BAP was not modified by GT treatment, our data clearly demonstrate an improvement of the hepatic redox state. This evidence might well be associated with the observed amelioration of the hepatic metabolic functions that are strongly dependent on the endocellular redox state [57]. Relevantly, the current experimental evidence demonstrates, for the first time in an in vivo model of MetS, the ability for GT to also reduce the HFD-dependent hepatic oxidative stress and lipid peroxidation. In line with previously reported evidence [58], our data show that HFD-induced obesity is associated with the development of hepatic macrovesicular steatosis. Although tomato supplementation has already been reported to both ameliorate hepatic steatosis and reduce the risk of Nonalcoholic Fatty Liver Disease (NAFLD) development in rats [59], no data are available on the impact of GT treatment on hepatic dysfunction. Interestingly, our results show for the first time that GT consumption significantly improves hepatic steatosis. These effects on the HFD-induced liver structural damage are consistent and in agreement with the ability of GT to improve the plasma lipoprotein profile and to relieve hepatic oxidative stress.

In our study, we also tested the effects of red tomato at full maturation in vivo on the redox homeostasis biomarkers, considering that the effect of this food matrix has already been studied on dysmetabolism [30,60]. On this point, we uncovered that treating MetS rats with RT for one month is able to partially improve the systemic antioxidant endogenous barriers impaired by HFD, such as by increasing BAP levels and returning to basal control values, though not acting on the thiolic groups. Indeed, RT extracts appear to selectively target plasma antioxidant barriers in HFD rats, which could be due to the fact that RT is richer in carotenes, molecules capable of strongly influencing the BAP test. The effect exerted by RT on BAP test is in line with the antioxidant power measured by CBA in the RT food matrix. In contrast, the oral administration of GT to HFD rats was able to enhance SHp levels, but does not influence BAP levels in accordance with the outcomes of FRAP assay.

Even more evidently, RT reduced the prooxidant status in terms of lypoperoxides and hydroperoxides. Similarly, the hepatic oxidative stress biomarkers were positively modified by RT treatment in terms of MDA, RONS, and GSH levels. All these outcomes evaluated with crucial parameters of redox homeostasis confirmed the specific effects of RT in MetS, in accordance with previous literature [61], supporting the importance of tomato-based products in the prevention of oxidant-driven dysmetabolism.

**Figure 10:**
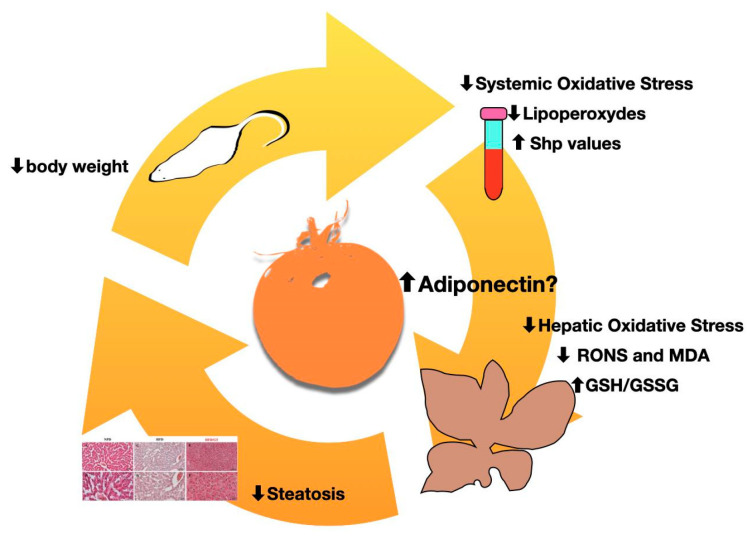
Schematic representation of possible pathophysiological processes implicated in the protection by GT in MetS. The protection exerted by GT on body weight gain, systemic, and hepatic oxidative stress levels (i.e., SHp, lipoperoxides, MDA, RONS and GSH/GSSG) could hypothetically be due to an increase in adiponectin.

A possible mechanism implicated in the GT-mediated protection exerted on HFD-induced liver damage could implicate adipokines (Figure 10). Indeed, alterations in adipokine levels are able to induce lipotoxicity and glucotoxicity in the liver with the development of steatosis through the involvement of TRP receptors [62]. Furthermore, according to data in the literature, naringenin—which we found to be specifically present in golden tomato—has been shown to act as a ligand for TRP receptors involved in many physiological processes that affect energy balance, inflammation, neuronal modulation, and oxidative stress [63,64,65]. It can be hypothesized that the reduction of free radicals observed in the liver and the regression of hepatic steatosis after treatment with the golden tomato could be associated with changes in the levels of the adipokines, leptin and adiponectin, as well as the involvement of TRP receptors, particularly TRPV1, as reported in the literature. This study opens up new possibilities for investigating the effect of golden tomato on the regression of NAFLD mediated by TRPV1 channels secondary to dietary activation of UCP2. Considering this, given the emerging clinical role of adipokines in cardiovascular disorders, the association of golden tomato with hypoadiponectinemia could suggest its role in the prevention of cardiovascular disorders.

Furthermore, it has been shown that there is a correlation between alterations in the gut microbiota and the development of NAFLD [66,67]. In this view, the intake of golden tomato, extremely rich in fiber, could lead to the production of acetic acid, butyric acid, and propionic acid (SCFAs) important in intestinal homeostasis. SCFAs produced by fiber fermentation are able to influence the regulation of intestinal hormones such as gut trypsin peptide, glucagon-like peptide-1 (GLP-1), leptin, and peptide tyrosine–tyrosine (PYY) involved in energy balance and to maintain the integrity of the intestinal barrier by reducing the entry of substances that can generate metabolic inflammation responsible for metabolic disorders [68,69,70]. The results of the present study provide also an opportunity to further investigate the effect of golden tomatoes on the gut microbiota, focusing also on pre/probiotic compounds that could play a role [71], and the possible preventive action of this food in metabolic syndrome.

## 5. Conclusions

Our research for the first time discloses the potential of “Golden Tomato” oral supplementation, via a comprehensive characterization of its biochemical, antioxidant, and disease-modifying properties, exploiting an in vivo rat model of MetS. The current investigation hints at GT as a powerful novel nutritional modulator and functional food, similarly to more widely known bioactive molecules that can also influence brain processes [72]. Indeed, in the context of nutritional supplementation and healthy diet, antioxidant-functional foods could underpin a robust strategy for the prevention and management of a wide plethora of physiological conditions, theoretically inasmuch as adaptative responses could be implicated in the homeostatic balance of complex networks [73]. This research hence substantiates the need for further investigations on tomato-based functional food, with a view to fully uncover GT nutritional application in food research.

## Figures and Tables

**Table 1:** Pellet composition is reported for both HFD and NPD. (SFA) Saturated Fat Acid, (MUFA) Monounsaturated Fatty Acid, (PUFA) Polyunsaturated Fatty Acid.

	Pellet HFD (PF4215)	NPDSND (PF1609)
Energy (Kcal/Kg)	5500–6000	3947
Fat Total (g/100 g)	60	3.50
SFA (g/60 g)	30	0.7
MUFA (g/60 g)	23	0.8
PUFA (g/60 g)	7	2
Crude protein (g/100 g)	23	22
Carbohydrates (Starch g/100 g)	-	35.18
Sugar (g/100 g)	-	5.66
Fiber (g/100 g)	5	4.5
Ash (g/100 g)	5.50	7.5
Vitamin A (IU)	8400	19.533
Vitamin D3 (IU)	2100	1260

**Table 2:** Analytical parameters of the two tomato samples expressed as percentages.

Analytical Parameters	Red Tomato	Golden Tomato
Moisture (% g)	94.2	91.5
Ash (%g)	0.8	0.9
Brix degree	5.8	5.3
Acidity (mg %)	0.5	0.6
pH	4.55	4.36

**Table 3:** Antioxidant components in tomato samples. The concentrations of golden and red (GT and RT) tomatoes are expressed as mg/100 g (Dried Weight, DW).

Antioxidant Components	Golden Tomato Concentration mg/100 gDW	Red Tomato Concentration mg/100 gDW
Vitamin C	170.4	311.2
𝛽-carotene	39.08	126.22
Lycopene	333.0	1971.0
Phytoene	6.5	16.8
Lutein	7.78	6.37
Naringenin	38.31	16.5
Gallic acid	2.0	<0.2
Chlorogenic acid	9.8	1.9
Rutin	4.68	7.1

**Table 4:** Biochemical parameters of lipid homeostasis triglycerides (TG), Total Cholesterol (TC), LDL Cholesterol, and HDL Cholesterol (mg/dL). Statistical significance for * *p* < 0.05 versus HFD and for ^#^ *p* < 0.05 versus NPD groups.

Experimental Groups	Triglycerides (mg/dL)	TC (mg/dL)	LDL Cholesterol (mg/dL)	HDL Cholesterol (mg/dL)
NPD	153.59 ± 61.49	60.90 ± 12.57	5.91 ± 3.15	34.35 ± 4.33
HFD	170.05 ± 57.44	86.57 ± 11.62 ^#^	14.08 ± 3.13 ^#^	30.02 ± 6.19
HFD/GT	183.58 ± 18.04	124.80 ± 23.12 *^,#^	9.98 ± 0.99 *^,#^	56.80 ± 9.01 *^,#^

## Data Availability

The data presented in this study are available on reasonable request from the corresponding author.

## Supplementary Materials

The following supporting information can be downloaded at: https://www.mdpi.com/article/10.3390/antiox12051121/s1, Table S1: Inclusion list of compounds sought and identified in the tomato samples analyzed; Table S2: Nutritional properties of tomato samples. The values are calculated on 100 g of edible part and are reported as the Mean ± SD of three repetitions; Table S3: Amount of micronutrients expressed in mg per 100 g of dry weight; Table S4: Quantity in mg of organic acids present in 100 g of dry product.
