# Supplementary material for: “Golden” Tomato Consumption Ameliorates Metabolic Syndrome: A Focus on the Redox Balance in the High-Fat-Diet-Fed Rat
Source: Antioxidants (Basel). 2023 May 18;12(5):1121. doi: 10.3390/antiox12051121 (PMC10215402; doi:10.3390/antiox12051121)
Supplement: Supplementary file 1 [file antioxidants-12-01121-s001.zip › antioxidants-2384293-supplementary.pdf]

Supplementary Experimental Section

2.1.2. Determination of phytonutrients in Golden and Red Tomatoes by HPLC system

Extraction phase of the analysis of phytochemicals in tomatoes

The extraction of phytonutrients has required a series of preliminary tests carried out in both tetrahydrofuran (THF) and dichloromethane/methanol at a 1/1 ratio. Tomato samples were dried and pulverized to allow better extraction of the phytonutrients. The samples were dehydrated in an oven at 65°C and subsequently pulverized with the aid of an electric grinder. The solvents tested for the extraction, dichloromethane/methanol at a 1/1 ratio and tetrahydrofuran, were chosen the first for its medium polar characteristics, while the second was used because it allows the immediate and complete dissolution of the components involved. Based on the recoveries of the substances, tetrahydrofuran was preferred.

Chromatographic conditions for the analysis of phytochemicals in tomatoes

Chromatographic separation was achieved on Luna PFP(2) (150 x 2.0 mm, 3 µm) equipped with precolumn, with 0.1% formic acid in water (mobile phase A) and 0.1% formic acid in methanol (mobile phase B).

A gradient method at 400 µL/min flow rate was applied as follows: start at 60% B, stay for 2 min; increase to 100% B over 8 min, held for 7 min; then decrease to 60% B over 2 min; maintained constant for 3 min. for a total run time of 20 min. Injection volume was 1 µL. A full mass/targeted SIM (t-SIM) scan methods were applied. The Orbitrap parameters were set as follows: alternate switching (-)/(+) ESI full scan mode and t-SIM, sheath gas flow rate 30 AU, discharge voltage 3.5 kV, capillary temperature 300 °C, resolution 35,000 FWHM, AGC target $5x10^6$, maximum injection time 200 ms and scan range 100–1000 m/z.

Table 1 reported below shows the compounds sought and identified in the tomato samples analyzed.

The precursor ions specified in the inclusion list are selected by quadrupole, fragmented in HCD cell with specific fragmentation energy and collected in C trap.

Table S1: inclusion list of compounds sought and identified in the tomato samples analysed.

Mass [m/z]	Chemical formula [M]	Species [z]	Polarity	Compounds
205.03625	$C_8H_{14}O_2S_2$	-H	Negative	a-Lipoic acid
271.06120	$C_{15}H_{12}O_5$	-H	Negative	Naringenin
536.43765	$C_{40}H_{56}$	+H	Positive	b-Carotene
545.50808	$C_{40}H_{64}$	+H	Positive	Phytoene
551.42474	$C_{40}H_{56}O_2$	[M + H - H_2O]	Positive	Lutein -H_2O

569.43531	$C_{40}H_{56}O_2$	+H	Positive	Zeaxanthin
609.14611	$C_{27}H_{30}O_{16}$	-H	Negative	Rutin

2.1.3. Determination of total polyphenolic content and antioxidant properties

Preparation phase of methanolic extract from fresh product

5 g of fresh tomato was weighed, homogenized in 20 mL of methanol and left to macerate for 24 hours. Finally they were filtered and made to a final volume of 25 mL with methanol (MeOH) obtaining a solution with a final concentration of 2%.

Total polyphenols by Folin-Ciocalteu (FC) assay

The total polyphenolic content has been evaluated using the Folin Ciocalteu assay with the use of a specific commercial kit and the Free Carpe Diem device (FREE® Carpe Diem; Diacron International, Italy). This method consists of the colorimetric determination of the total polyphenols (TP) and it is based on the oxidation of the phenolic compounds by the Folin-Ciocalteu (FC) reagent which contains phosphomolybdic/phosphotungstic acid complexes. In particular, it relies on the transfer of electrons from phenolic compounds to form a blue chromophore where the maximum absorption depends on the concentration of phenolic compounds in the sample and which is spectrophotometrically detectable at 630 nm. Gallic acid has been used as the reference standard to create the calibration curve. For the evaluation of TP it has been necessary to prepare a blank with 1,5 ml of FC reagent and 25 ml of distilled water and each sample with 1,5 ml of FC reagent and 25 ml of sample. After incubating for 5 minutes at 37 °C the absorbance has been measured. The data have been expressed as gallic acid equivalents (mg/L).

Ferric Reducing Antioxidant Power (FRAP) Assay of Tomatoes

The FRAP method measures the reduction of the ferric complex 2,4,6-tripyridyl-s-triazine $[Fe^{3+}\text{-}(TPTZ)_2]^{3+}$ to the ferrous complex [deep blue ferrous complex $[Fe^{2+}\text{-}(TPTZ)_2]^{2+}$ in an acid environment[1]. A Beckman spectrophotometer, model DU 640, was used for the analysis. FRAP values are obtained by comparing the absorbance change at 593 nm in samples with blank. The FRAP working reagent was prepared daily, according to [1], by adding to ten parts of sodium acetate (CH_3COONa) at pH 3,6, one part of ferric chloride (FeCl3) and one part of 2,4,6-Tripyridyl-S-Triazine (TPTZ) soluble in HCl 40 mM. For the assay, it has been necessary to prepare a blank with 1,5 ml of FRAP reagent and 200 ml of distilled water and the samples with 1,5 ml of FRAP reagent and 200 ml of each sample. All procedures have been carried out away from direct exposure to light.[1,2].In order to make the results comparable with what is in the literature, an average calibration line was constructed with Trolox in the concentration range of 0.04 to 0.1 mM, and the results were expressed in mM Trolox taking into account the dilution factor.

Radical Scavenger activity of Tomatoes by Crocin Bleaching Assay (CBA)

The CBA was applied to several food matrices as suitable for screening radical scavenging activity. It has an advantage over the others, in that it is able to detect either the antioxidant or the pro-oxidant action of the compound or mixture under analysis. The method was performed on lyophilisate samples. Crocin was extracted twice from authentic commercial saffron (origin grade) according to a validated protocol and the estimation of its concentration to ~3 μM was based on an extinction coefficient reported in the literature, εMeOH = 1.33 × 105 mol−1 cm−1. Crocin working solutions were daily prepared in methanol (Merk) so that after adjustment the A443 value was ~3.9. A certain volume of crocin working solution was diluted with methanol to 25 mL (total volume) so that the A_{443} value was ~1. Then a 2,2′-azo-bis(2-aminopropane)dihydrochloride], APAB, purchased from Wako Chemicals, stock solution (12.5 mM) in distilled water was prepared. Finally, the reaction mixture, in the presence of tomato, consists of 0.15 mL of APAB, 0.15 mL of diluted crocin and increasing amounts (0.15-0.02mL) of tomato extract to the final volume of 1mL of distilled water. The reaction is conducted at a thermostatically controlled temperature of 37°C and starts after the addition of the radicalizing agent (APAB). Two minutes are waited to give the system time to equilibrate and consists of monitoring the kinetics of crocin bleaching for 10 minutes. using a Beckman 640 UV/visible spectrophotometer, against a blank. A "blank" is a solution composed of the same reagents without crocin.

Expressing results

According to competition kinetics, the rate of crocin bleaching in the presence of lipoperoxide radicals (V0) decreases in the presence of antioxidants to a value of Va, in our case the antioxidant being tomato extracts. Various levels of tomato (0.15–0.02 mL) were also tested so that linear regression curves of relative rates (Δ*V*0/Δ*V*a) against the (tomato samples/crocin) ratios could be built.

The five-point linear regression slopes representing the relative rate constants (*K*rel = *K*a/*K*c) were calculated according to the following formula

$$\frac{Vo}{Va} = 1 + \frac{Ka}{Kc} * \frac{[A]}{[C]}$$

where *K*a is the rate constant for the reaction between antioxidant and peroxyl radicals; *K*c is the rate constant for the reaction between crocin and peroxyl radicals; [*A*] is the concentration of tomato samples and [C] is the concentration of the crocin. All the concentrations are expressed as %v/v (volume-to-volume percentage). The value of the ratio *K*a/*K*c (Keq) indicates the relative capacity (antioxidant capacity) of tomato samples to interact with peroxyl radicals.

V0 is the rate of the reaction of the crocin with peroxyl radical calculated as follows:

V0= [Abs_{443}(t=2min) -Abs_{443}(t=12 min)] crocin without tomato;

Va is the rate of the reaction of the crocin with peroxyl radical in presence to different levels of tomato extract (0,15-0,02mL) and is calculated as:

Va $=\Delta\ [Abs443(t = 2min.) - Abs443(t{=}12\text{min.})]$ for each concentration (%v/v) of tomato samples

By dividing the ($K_{rel} = K_a/K_c$) of tomato samples by the K_{rel} value of a millimolar concentration of Trolox, equivalent to 9mM, we obtain a ratio between the rate constants; this value represents the antioxidant capacity expressed as Trolox equivalents (TRE). We express the results as TRE.

3. Supplementary Results

3.1. Analytical, nutritional and antioxidant composition of Tomato food matrices

Table S2. Nutritional properties of tomato samples. The values are calculated on 100 grams of edible part and are reported as the Mean $\pm SD$ of three repetitions

Parameters	Golden Tomato	Red tomato
Edible part (%)	100	100
Energy (Kcal)	15	17
Kjoule	63	71
Moisture (g)	91.47±2.37	94.2±1.34
Ash (g)	0.9 ±0.1	0.8 ±0.2
Total Protein (g)	1.28±0.07	1.36±0.10
Lipid (g)	0.16±0.04	0.19± 0.05
Carbohydrate (g)	2.13±0.12	2.44±0.07

Table S3. Amount of micronutrients expressed in mg per 100 g of dry weight.

Mineral compounds	Units	Golden Tomato	Red Tomato
Sodium (Na)	mg/100g	90.9	169.9
Potassium (K)	mg/100g	930.1	1451.8
Calcium (Ca)	mg/100g	277.4	122.5
Zinc (Zn)	mg/100g	6.7	2.3
Iron (Fe)	mg/100g	37.9	5.8
Copper (Cu)	mg/100g	4.03	1.2
Nickel (Ni)	mg/100g	0.24	0.1
Manganese (Mn)	mg/100g	1.7	1.0
Alluminium (Al)	mg/100g	95.16	13.77

Table S4. Quantity in mg of organic acids present in 100 g of dry product.

Organic acids	Chemical formula	Golden Tomato mg/100g	Red Tomato mg/100g
Mali acid	C_4H_6O	1040	520
Citric acid	$C_6H_8O_7$	6840	6560
Tartaric acid	$C_4H_6O_6$	520	600
Ossalic acid	$C_2H_2O_4$	80	< 10

References

[1] I. F. Benzie, J. J. Strain, *Methods Enzymol.* **1999**, *299*, 15–27.
[2] İ. Gulcin, *Arch. Toxicol.* **2020**, *94*, 651–715.
